# Appendage regeneration is context dependent at the cellular level

**DOI:** 10.1098/rsob.210126

**Published:** 2021-07-28

**Authors:** Can Aztekin

**Affiliations:** School of Life Sciences, Swiss Federal Institute of Technology Lausanne (EPFL), 1015 Lausanne, Switzerland

**Keywords:** appendage regeneration, cross-species, regeneration, single-cell-omics, cellular level

## Abstract

Species that can regrow their lost appendages have been studied with the ultimate aim of developing methods to enable human limb regeneration. These examinations highlight that appendage regeneration progresses through shared tissue stages and gene activities, leading to the assumption that appendage regeneration paradigms (e.g. tails and limbs) are the same or similar. However, recent research suggests these paradigms operate differently at the cellular level, despite sharing tissue descriptions and gene expressions. Here, collecting the findings from disparate studies, I argue appendage regeneration is context dependent at the cellular level; nonetheless, it requires (i) signalling centres, (ii) stem/progenitor cell types and (iii) a regeneration-permissive environment, and these three common cellular principles could be more suitable for cross-species/paradigm/age comparisons.

## Graphical abstract

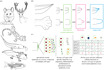

## 1. Introduction

**Figure 1.**
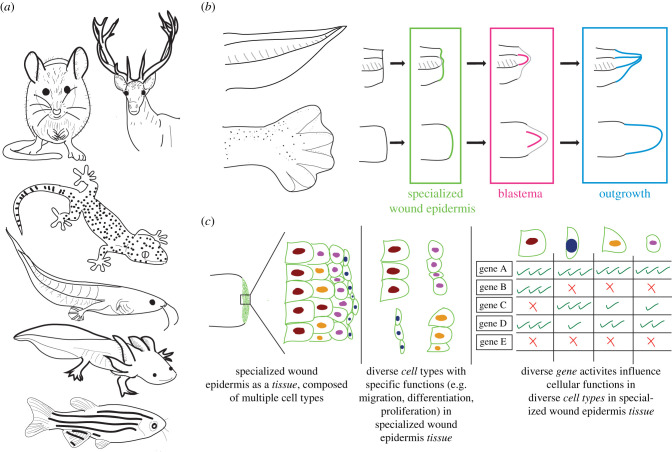
Appendage regeneration paradigms share similar tissue descriptions, but the cellular composition of these tissues and similarities across paradigms remain unclear. (*a*) Multiple widely used model organisms can regenerate different appendages. These include (from top to bottom): mouse digit tips; deer antlers; lizard tails; early-stage tadpole limbs and tails; axolotl limbs, digit tips, tails and gills; zebrafish fins. (*b*) Appendage regeneration in different species and paradigms progresses through three common tissue stages, and schematics exemplifying these stages are shown during tail (top) and limb (bottom) regeneration. (*c*) Appendage regeneration is mostly characterized at the tissue and gene level. Meanwhile, tissues are composed of multiple cell types with distinct specific functions (e.g. differentiation, proliferation) controlled by diverse and mostly non-specific gene activities.

Regenerative medicine aims to identify approaches to enable the replacement of lost or damaged organs. These efforts include the potential for human limb regeneration by studying certain species (e.g. salamander, zebrafish and mouse) that can regrow their lost appendages (e.g. limb, tail and digit tip) (figure 1*a*). Appendage regeneration scenarios follow distinct tissue-level stages [1,2] (figure 1*b*). Briefly, upon amputation, these animals cover their amputation plane without a scar and then form a specialized wound epidermis (also known as apical epithelial cap, AEC) [3] to modulate the growth of underlying tissue by contributing to various cellular mechanisms. Afterwards, owing to their interaction with the specialized wound epidermis, a tissue type called a blastema is induced [4]. Cells in a blastema serve as the building blocks of the regenerated appendage. Finally, the interaction between the specialized wound epidermis and blastema enables the outgrowth phase and restores the lost appendage. Due to the similarities in tissue morphologies (e.g. thickened wound epithelium, morphologically undifferentiated looking cells in a blastema), the usage of some shared gene expressions (e.g. *Fgf20* in specialized wound epidermis [5,6], *Prrx1* in blastema [7–9]) and the requirement for specific signalling pathway activations (e.g. FGF pathway [10,11]), appendage regeneration has been considered effectively as a singular programme responsible for different regeneration paradigms (e.g. tails, limbs, digit tip, gills and fin) irrespective of species and for different age groups. This assumption makes comparisons feasible, and even enables assessments between appendage versus non-appendage regeneration paradigms, such as comparing limb regeneration to planaria whole-body regeneration or mouse hair regeneration. However, the lack of available approaches to examine cell types in highly heterogeneous tissues hindered our understanding of whether indeed similarities at the tissue and gene level are also reflected at the cellular level; if not, what are common *cellular* principles guiding appendage regeneration across species and paradigms?

The bulk of our understanding of appendage regeneration relies on tissue-level descriptions and studies linking systemic genetic perturbations to tissue-level observations. However, genes do not operate alone, and genes do not directly affect tissues; instead, genes operate within cells and affect cells that compose tissues (figure 1*c*). Moreover, the same gene may show different effects depending on the investigated cell type and the regeneration paradigm. For example, during limb regeneration, *Shh* is expressed in the mesenchymal cells and mediates digit patterning [12–15]; during tail regeneration, *Shh* is expressed in spinal cord and notochord and essential for their proliferation [14,16]; during fin regeneration, *Shh* is expressed in basal epidermal cells and enables bone maturation [17]. Critically, regeneration involves multiple coordinated cellular mechanisms (e.g. proliferation, migration and differentiation), and depending on the cell type, each of these cellular mechanisms may involve distinct gene activities (figure 1*c*). Hence, it seems very unlikely that regeneration is run by a single programme that is seen in the cells of the whole animal. Therefore, collectively, it is of utmost importance to decipher how gene activities affect individual cells, cell types and cellular mechanisms, and how this impacts tissues and regeneration.

Recent years have provided exciting opportunities to tackle certain technical limitations in vertebrate regeneration research. These include single-cell multi-omics methods (e.g. single-cell RNA-sequencing (scRNA-Seq)), less-toxic high-content live-imaging platforms and the CRISPR-Cas9 system for feasible gene editing. These innovations challenge long-used descriptions and started deducing the tissue-level observations that underlie the cellular mechanisms. Moreover, recent reports suggest that not all vertebrate regeneration scenarios use the same cell types and cellular mechanism. Regeneration paradigms (e.g. tail versus limb), species (e.g. newt versus axolotl) and age of the animals may involve different cell types and cellular mechanisms. Such differences could be beneficial as they can open multiple paths for mammalian limb regeneration but also warrant caution for cross-species comparisons. Due to this, here, I showcase the phenotypic richness within vertebrate appendage regeneration scenarios to highlight context dependency at the cellular level. As the diversity within vertebrate appendage regeneration scenarios already underscores a high degree of context dependency at the cellular level, I will not compare these paradigms to invertebrate regeneration models (e.g. planaria) or to other organ regeneration models (e.g. heart regeneration). Finally, by examining common cellular themes, I hypothesize that, although cell types may change depending on the context, there are three broad cellular components for vertebrate appendage regeneration—(i) signalling centres, (ii) stem/progenitor cell types and (iii) regeneration-permissive environment—and that these three principles could be more suitable for cross-species comparisons.

## 2. Signalling centres

Appendage regeneration requires signalling centres, populations influencing cellular phenotypes and leading to morphological changes by expressing high and varying mitogenic, chemotactic and inductive signals. These signals then influence the surrounding cells for proliferation, migration and cell-fate decisions. A prominent example of a signalling centre during regeneration is the specialized wound epidermis, the apical epithelial cap (AEC). It has long been hypothesized that higher vertebrates, including humans, fail to form this tissue; hence regeneration fails [18]. Although there was no precise cellular and molecular definition of the specialized wound epidermis across all regeneration paradigms, they have been mostly characterized by their appearance at the amputation plane and as the absolute requirement for regeneration. Preventing the formation of the specialized wound epidermis in salamander limb or *Xenopus* tail regeneration inhibits the growth of a new appendage [4,19,20]. Meanwhile, the elimination of other cell populations involved in regeneration can only perturb the regrowth of the structure. For example, removal of a subset of digit tip fibroblasts results in impaired regeneration, yet some growth is still seen [21]. Likewise, the elimination of melanocytes [22], perturbing muscles [23] or even removal of the spinal cord [24] is not sufficient to completely block proliferation at the *Xenopus* tail amputation plane. Likewise, perturbing *Pax3* in newts results in limb development without muscles, and when their limbs are amputated, they can still regrow a limb structure without muscles [25]. Complementing their high secretory profile, grafting tissues that contain the specialized wound epidermis induces ectopic outgrowths, further exemplifies their ability to induce proliferation of underlying tissue [19,26–28]. Although the specialized wound epidermis is essential for regeneration, the cellular composition of this structure was not clear, yet assumed similar in different regeneration paradigms.

The specialized wound epidermis, the AEC, was mostly studied in the context of salamander limb regeneration. Before examining further, it should be noted that the wound epidermis and AEC do not indicate the same tissue and the definitions segregating these two have been previously reviewed [3]. Briefly, the wound epidermis was defined as the epidermal tissue covering the wound following amputation, meanwhile, a morphologically identified thickened epithelium at the amputation plane associated with regeneration was used to define AEC [3]. More recently, molecular markers were identified (e.g. *Fgf8*) to detect the AEC [29,30]. As both the AEC and apical-ectodermal-ridge (AER), which forms during limb development, were suggested to be required for proximal-distal axis elongation and share some marker gene expressions, they were considered to be analogous tissues [1,3]. However, there were several problems with this comparison. First, the AEC has been predominantly characterized by studying salamander limb regeneration; however, salamander limb development occurs without an observable AER structure [31,32]. Conversely, the AER is mostly characterized in amniotes [33] and found to be in humans [34,35]) that are limb regeneration-incompetent and do not have AEC. Therefore, the assumption AER and AEC are analogues relies on characterizations that are conducted in a mutually exclusive manner. Second, apart from a few marker genes, there was no high-throughput and unbiased comparison between these two tissues, and individual genes may be used for different purposes in different cell types. Moreover, recent studies of axolotl limb regeneration resulted in inconsistent findings for *Fgf8* expressing epidermal populations specific for regeneration [13,30,36–40]*.* Third, the cellular composition of skins is significantly different for pre- and post-metamorphic amphibians, and embryonic and adult amniotes, bringing additional challenges to compare cells defining AEC and AER [9,41–43]. Due to these limitations, it has remained unclear if the AEC results from a re-deployment of AER developmental programme or a novel cell state specific for regeneration. Identifying similarities between cells populating the AEC and the AER impacts our understanding of the potential for human limb regeneration. If the AEC and the AER are composed of the same cell type, then mammals would have the transcriptional programme for regeneration, but fail to activate it. If the AEC is a novel cell state, then mammals would lack the transcriptional programme encoded in the genome for this cell type. *Xenopus* is the only commonly used model organism that has a clearly identified developmental AER and limb regeneration-associated AEC [31,44]. By using *Xenopus* and scRNA-seq, the cellular composition of the AEC and the AER tissues and their molecular similarities were found to be almost identical [45]. This suggests that the AEC is a re-deployment of the developmental AER transcriptional programme. Nonetheless, there seems to be no conservation of the cellular characteristics of the specialized wound epidermis across all regeneration paradigms.

**Figure 2.**
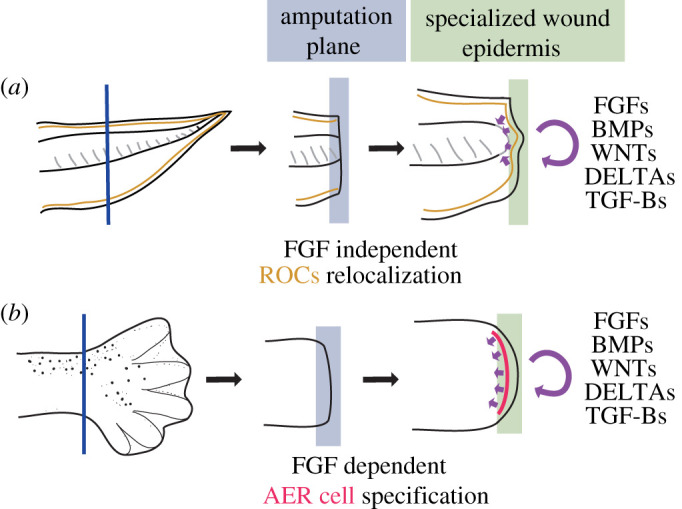
*Xenopus* tail and limb specialized wound epidermis tissues are composed of different cell types and form via different cellular mechanisms. (*a*) *Xenopus* tail regeneration uses ROCs (yellow) that act as a signalling centre defining the specialized wound epidermis. ROCs are found in the tadpole tail before amputation and relocalize from the posterior trunk to the amputation plane upon amputation. ROC relocalization does not depend on FGF receptor activity. Meanwhile, (*b*) *Xenopus* limb regeneration uses apical-ectodermal-ridge (AER) cells (pink) to act as a signalling centre defining the specialized wound epidermis. AER cells are not found in the tadpole limb before amputation and are specified upon amputation. AER cell specification depends on FGF receptor activity.

Recent studies have demonstrated that the specialized wound epidermis shows differences across regeneration paradigms. First, a morphologically thickened multilayer of specialized wound epidermal tissue, as in salamanders, is not necessarily seen during zebrafish fin [46,47], *Xenopus* tail [48], or mouse digit tip regeneration [49,50]. Second, molecular markers that are commonly used in limb regeneration (e.g. *Fgf8, Msx2 *and* Wnt5a* [51]) may be lacking in different appendage regeneration scenarios. For example, *Fgf8* is absent in the wound epidermis of the amputated zebrafish fin [52], although it expresses another genes associated with the wound epidermis. Recently, an epithelial cell type that is termed regeneration-organizing cells (ROCs) was found to be the cell type defining the specialized wound epidermis during *Xenopus* tail regeneration [19]. Afterwards, ROCs and AER cells were found to have differences in their transcriptome, single-cell morphology and cellular mechanisms [45]. Specifically, while ROCs are present in the body before tail amputation and their relocation to the amputation plane is critical for regeneration [19], AER cells have to be specified during regeneration [45] (figure 2). While FGF inhibition does not significantly affect ROCs relocalization to the tail amputation plane to act as a specialized wound epidermis [19], it impairs AER cell specification during limb regeneration [45] (figure 2). Although both ROCs and AER cells elicit the properties of the specialized wound epidermis, they do not compose the whole epidermal tissue at the amputation plane (figure 2). Instead, they can be found only at the basal layers of the amputation plane epidermis, highlighting the cellular heterogeneity within the specialized wound epidermis tissues. Despite their differences, transcriptional similarities between these cell types may hint at the existence of a core gene regulatory network necessary for signalling centre abilities. However, it remains unclear if ROCs or AER cells are present in other systems or if different cell types exhibit a signalling centre transcriptome during different appendage regeneration paradigms. Similar single-cell transcriptome and cellular mechanism-based comparisons between species and paradigms could reveal similarities between cell types defining the AEC.

Apart from the epithelium at the amputation plane, other populations may also act as a signalling centre influencing regenerative outcomes. During mouse digit tip regeneration, nail bed stem cells serve as a signalling centre and are required for regeneration [53,54], although they do not form at the amputation plane. On another note, limb regeneration in amphibians, much like limb development, involves multiple signalling centres. In addition to an AER, it also requires re-establishment of the zone of polarizing activity, ZPA [55] (acting as a signalling centre by expressing *Shh*) [56,57], to pattern an autopod with digits. Meanwhile, other regeneration scenarios such as tail or digit tip regeneration do not have digit patterning and do not form a ZPA. Hence, these differences highlight that different regeneration paradigms (e.g. limb versus digit, limb versus tail) do not have a requirement for the same set of transcriptional programmes.

There may be unknown signalling centre cell types. Some appendages that are heavily composed of cartilaginous tissue, commonly do not regenerate with a known epithelial signalling centre. The existence of a specialized wound epidermis for deer antlers that are primarily composed of chondrogenic lineage cells is debated [58]. Likewise, late-stage *Xenopus* tadpoles can grow a cartilaginous spike upon amputation without forming a specialized wound epidermis [29,59]. Lastly, without having a distinct specialized wound epidermis, the mouse digit tip model system heavily relies on bone growth [60]. Altogether, these results suggest that cartilaginous appendages can elongate and create specific structures without epithelial signalling centres forming at the amputation plane. Nonetheless, it remains unclear if cartilaginous tissues are regulated by different mechanisms upon injury, such as gaining mesenchymal signalling centre properties. Indeed, recently, mouse rib regeneration was suggested to be organized by a signalling centre *Sox9*+ mesenchymal population [61], and it is possible that chondrogenic lineage itself may be showing similar features during appendage regeneration scenarios.

Genes associated with signalling centres may be expressed from different cellular sources between species. For example, in the axolotl, AER cell-derived signals (e.g. FGFs) are secreted from their mesenchyme instead of the epithelium (a detailed comparison can be found in Purushothaman *et al*. [31]). The change in the gene expression pattern and cellular source of ligands can impact the absolute number of signalling ligands and potentially forming morphogen gradients within a limb. Distal epithelium-derived morphogen gradients would form from distal to proximal fashion, while mesenchyme-derived FGFs would not form such a gradient. These types of differences between species may enable mesenchymal cells to act as signalling centres and could be a key factor contributing to the axolotl limb regeneration to be a more persistent ability. Overall, signalling centres are essential for the outcome of appendage regeneration. Nonetheless, these centres do not need to be the same cell type, be present at the same location, nor exhibit the same cellular mechanisms to instruct the building blocks (stem/progenitor cell types) to reconstitute the lost structure in the correct manner.

## 3. Stem and progenitor cell types

As in every development, the new appendage also requires stem and progenitor cells. In this review, I refer to stem cells as populations that can self-renew and differentiate, and progenitor cells as transiently formed groups of cells with a lesser differentiation capacity. These cells are found in the blastema tissue during regeneration. Moreover, any cell found in blastema is referred to as a *blastema cell* and their pre-amputation progeny as potential *blastema progenitor*. The definition of blastema has changed considerably in the last century. However, in all these descriptions, the requirement of *stem/progenitor cell types* has remained an essential component.

Historically, blastema was suggested to be composed of a pluripotent homogeneous cell type formed through dedifferentiation of somatic cells. This definition, by default, also suggested the existence of a regeneration-specific blastema cell type which could be seen across species and paradigms. These proposals were partly due to findings advocating that tissues, such as, skin [62] or nerves [63] can form all components of the regenerated limb. However, these studies, which were conducted by grafting and lineage-tracing cells, were littered with different technical caveats and parameters that were difficult to control, hence affecting interpretations (discussions on this topic can be found elsewhere [64,65]). For example, the cellular composition of tissue grafts may not be known and could include contaminant cell types influencing the interpretations. Additionally, preparation of the grafts involves an injury which can impact cellular plasticity; culturing cells *in vitro* prior to grafting, and the grafting method itself can alter cellular behaviours. Similarly, transplanted new microenvironment (e.g. proximity to certain populations) and transplanted new macroenvironment (e.g. animal developmental or metabolic state) could affect cellular programmes. Such caveats have been brought to light during the last decades, partly due to the usage of complementary molecular tools, as well as novel insights gained from mechanobiology studies. Hence, such historical findings emphasize the critical need to assess cell-fate changes *in situ* and via complementary methodologies.

In recent years, research involving various molecular tools found that the blastema does not contain pluripotent cells, but rather contains heterogeneous lineage-restricted stem/progenitor cell types [50,66–69], without necessarily invoking the existence of dedifferentiated cells (as seen with *Pax7*+ satellite cells, muscle stem cell-like cells [23,70,71]). These findings also stress that it is very unlikely that there is a regeneration-specific blastema cell type shared across all paradigms. As the composition of appendages (e.g. tail, limb, fin, digit tip) considerably differs, instead of one type of a blastema cell, different stem/progenitor cell types would be required for different appendage regeneration scenarios. For example, notochord is not found at the tips of digits. Hence, a digit tip blastema would not require notochord progenitor cell types. Moreover, even the same type of appendage (e.g. limb) can show differences between species for its cellular composition and developmental mechanisms guiding their formation [31,44,72]. Investigating which stem/progenitor cell types are being used for appendage regeneration scenarios can encourage the identification of counterparts in humans to test if our cells have the competency to participate in regeneration.

**Figure 3.**
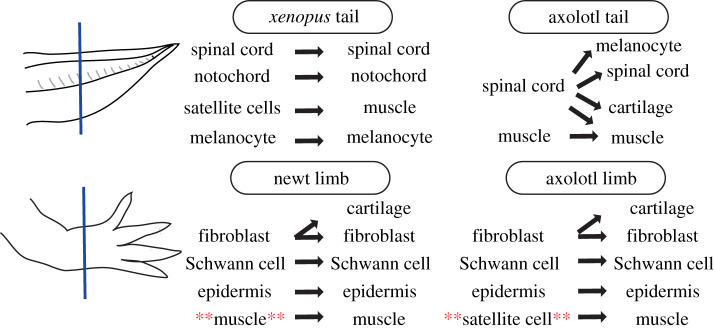
There is no one type of blastema cell type across paradigms, and appendage regeneration uses different lineage-restricted stem and progenitor cell types to re-build the lost appendage. Different stem and progenitor cell types, with different cellular potencies, are used depending on species or the regeneration paradigm. Red asterisks are added to the text to highlight the difference between a newt and an axolotl limb. Studies used to describe examples in this figure are as follows: *Xenopus* tail [23,66]; axolotl tail [74,75]; newt limb [71,76]; and axolotl limb [67,70,77].

Muscles are one of the crucial tissue types present in most regenerated appendages. Early research suggested that muscles dedifferentiate from terminally differentiated multinucleated muscle fibres to mononucleated muscle progenitors to regenerate the limb [73]. However, more diverse cellular mechanisms were documented to be present depending on species, age and regeneration paradigms. While axolotls (*Ambystoma mexicanum)* use *Pax7+* satellite cells (adult muscle stem cells, cells retaining stem cell features after embryonic development) to regenerate muscles in a limb, newts (*Notophthalmus viridescens*) use dedifferentiation for the same purpose [70,71]. In addition to species, age also influences the cellular mechanisms used for regeneration. To generate new muscles in the limb following injury, post-metamorphic newts (*Cynops pyrrhogaster*) use dedifferentiation as the major mechanism to generate a source of cells for muscle regeneration (figure 3); however, during pre-metamophic stages *Pax7+ *satellite cells are used** [76] (figure 3). In another regeneration paradigm, *Xenopus* tail regeneration, embryonic *Pax7+* satellite cells, rather than muscle dedifferentiation, were suggested to be the mechanism used to regenerate muscles [23,78]. Meanwhile, axolotl tail regeneration was recorded to use muscle dedifferentiation [74]. Humans also have *Pax7+* satellite cells aiding in muscle-injury and homeostasis [79]. Investigating if these satellite cells are similar across species, how these cells are activated in animals, and whether they can be activated in the same way in humans for limb regeneration, would be an exciting area to explore. Overall, regeneration can involve the contribution of *adult or embryonic* stem/progenitor cell types.

How stem and progenitor cells are formed upon amputation and differentiate into necessary lineages during regeneration remains a stimulating research topic. Recent research mostly focused on the origin and fate of cells in a blastema. These results suggested that salamander limb regeneration involves lineage-restricted stem/progenitor cells including epidermal, muscle and Schwann cells [67,76] (figure 3). Moreover, tissue-tracing [67,76,77,80], genetic lineage-tracing [81] and scRNA-Seq-based computational prediction [36,81] approaches demonstrated that axolotl limb regeneration uses multipotent mesenchymal cells that can differentiate into cartilage and fibroblasts (figure 3). Meanwhile, early research suggested that a certain amount of injury during newt limb amputation induces dedifferentiation of cells that will be used in regeneration [4]. In these studies, dedifferentiation was considered as a change to a more circular cell morphology, cells with a higher nucleus/cytoplasm ratio and the ability to re-enter to cell-cycle [82]. Subsequently, the formed AEC sends signals enabling maintenance and proliferation of these dedifferentiated cells to create the blastema. In the absence of the AEC, dedifferentiated cells can still be observed but disappear after some time [4]. Although our understanding of lineage relations during regeneration has expanded in recent decades, it remains largely ambiguous how injury relates to the acquisition of mesenchymal cell multipotency and how the AEC directs the cells to regenerate an appendage.

**Figure 4.**
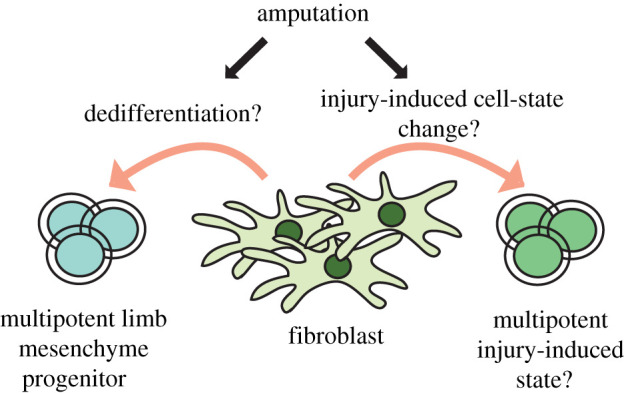
Acquisition of a more plastic cell fate: dedifferentiation versus injury-induced states. Dedifferentiation is considered as the cell-fate change from a terminally differentiated cell to a more primitive, potentially multipotent, developmental cell type. Recent single-cell RNA-sequencing results suggest amputations might be inducing terminally differentiated cells to acquire an injury-induced, potentially multipotent, cell state resembling developmental cell types, rather than resetting to a developmental cell type.

True regeneration-specific events can be distinguished from injury responses by comparing species that have both regeneration-competent and regeneration-incompetent conditions. Mouse digit tip regeneration depends on the amputation level [49,54], and *Xenopus* limb regeneration depends on the amputation level [83] as well as the developmental stage [59]. Recent work by comparing regeneration-competent and -incompetent conditions in these model systems by single-cell transcriptomics showed that, independent from the regeneration outcome, amputations induce a subset of fibroblasts to express genes associated with blastema [21,45], highlighting that gene thought to be regeneration-specific previously, may constitute a response to injury. Moreover, it has been shown that injury to the upper arm of a *Xenopus* and axolotl, under certain conditions, or electroporation of newt limb or tail can also induce morphologically identified dedifferentiated cells resembling cells in a blastema [84–87]. These findings may align with earlier work in salamanders implying that a certain amount of injury is the inducer of morphologically identified dedifferentiation, as a process of cell-fate change from a differentiated cell type to a primitive developmental progenitor cell type. However, results from transcriptomics approaches challenge the notion of dedifferentiation. Is morphologically identified fibroblast dedifferentiation corresponding to complete conversion to a more primitive developmental cell state (a multipotent limb mesenchyme progenitor cell type), or is this phenotype an injury-induced novel cell state (figure 4)? If the first is true, gene regulatory networks are re-wired to developmental states and should recap developmental functions. Meanwhile, the second possibility would indicate establishing new gene regulatory networks that are not observed during development and potentially exhibit new functions. Based on the latest single-cell transcriptomics results from different species (*Xenopus* [45,88], mouse [21] and the axolotl [80]), specific fibroblast cells express genes associated with blastema and show transcriptional programmes that are not seen during limb development. More specifically, in froglets, amputation was shown to induce a subset of fibroblasts to initiate a chondrogenic transcriptional programme that is different than the chondrogenic programme during limb development [88]. Hence, the previous terminology labelling so-called dedifferentiated cells could be indicating an injury-induced cell state for some fibroblast cell types rather than resetting to a developmental cell state. Overall, not just stem/progenitor cell types, but also *injury-induced* cell-fate changes may be involved in regeneration.

Cell-fate changes crossing germ layers, transdifferentation, are not necessarily observed in all regeneration paradigms. For example, lineage-restricted stem/progenitor cells were suggested to mediate zebrafish fin, and *Xenopus laevis* tail regeneration: the spinal cord produces a new spinal cord, but the spinal cord does not restore notochord or muscle tissues [66,69] (figure 3). Single-cell transcriptomics results further support these conclusions that there is no multipotent cell type exhibiting multiple germ layer associated gene expressions during *Xenopus* tail regeneration [19,89]. Nonetheless, these results do not omit a possibility of dedifferentiation for nervous system or notochord cells to regenerate a tail. As an example, terminally differentiated neurons may acquire a spinal cord progenitor identity during regeneration. Indeed, dedifferentiation while maintaining lineage restriction was observed for osteogenic cells and Schwann cells during zebrafish fin and mouse digit tip regeneration, respectively [69,90–92]. These results indicate that not all appendage regeneration paradigms generate and use multipotent cell types. On the other hand, tail regeneration in axolotl and lizard was suggested to involve transdifferentiation, presumably through multipotent cell types. In lizards, cartilage to/from muscle lineage [93], and in axolotls, spinal cord to mesodermal lineage [75], were recorded as possible transdifferentiation events (figure 3). However, the axolotl phenotype was later suggested to be predominantly seen in specific experimental set-ups [94] stressing an additional layer of complexity of cellular behaviours upon perturbations.

Altogether, with contemporary advances, it is evidenced that the species, regeneration paradigm and age of animals influence the cell types being used as well as cellular mechanisms to build a blastema. Unlike earlier suggestions, there is no pluripotency during vertebrate appendage regeneration; most lineages seem to be reconstituted from their own. Overall, to build a new appendage, it is crucial to either have stem/progenitor cell types before amputations or involve cellular mechanisms that allow cells to expand their lineage and cell division potential.

## 4. Regeneration-permissive environment

As well as specific cell types, a regeneration-permissive environment at the amputation plane is also critical for appendage regeneration. Here, I refer to the regeneration-permissive environment as the cellular composition of the amputation plane, state of the extracellular matrix and available secreted factors enabling signalling centres and stem and progenitor cells to form and interact with each other. Salamander limb regeneration is studied in laboratories by amputating limbs with a near-vertical angle and result in near-perfect regeneration, while limb loss via predation and amputations with varying angles typically result in imperfect regeneration [95,96]; repeated amputations to salamander limbs can result in defects [97,98]; *Xenopus* tadpole limb regeneration becomes problematic for proximal limb amputations compared to distal—similarly for amputations through the cartilage/bone compared to those through the joint [83]; a mouse can only regenerate distal digit tips, while proximal amputations result in failed regeneration [49]. Such differences in position-dependent amputation outcomes could be due to the homeostatic states of appendages. Amputations practically destroy a highly organized structure and result in a disordered state. Hence, changes in the extracellular matrix (ECM), tissue composition near the amputation plane, and secreted factors are likely to influence signalling centres and stem/progenitor cell types.

The activity of the immune system is critical to setting the regeneration-permissive environment. Species with lower regenerative abilities tend to have a more sophisticated immune system compared to regeneration-competent ones [99]. Due to this, it has been speculated that a strong immune response might have been one of the causes of the regeneration incompetency and reduced regenerative abilities. Regeneration-competent species are considered to suppress amputation-induced inflammation much more efficiently [100]. Meanwhile, regeneration-incompetent species exhibit sustained inflammation, and amputations in these animals result in fibrosis and scar formation. Interestingly, interference to regulatory pathways related to the immune system (e.g. reactive oxygen species [101–104], hydrogen pump activity [105] and oxygen influx [106]) can determine the regeneration outcome. Successful appendage regeneration, so far, has been shown to require the presence of macrophages (more broadly myeloid lineage) in a vast selection of regenerative vertebrates through their impact on the changes in ECM (zebrafish fin [107,108]; axolotl limb [109]; mouse digit tip [110,111]; lizard tail [112]; and *Xenopus* tail [113]). Additionally, amputations induce apoptosis as well as cellular senescence, and their clearance via immune cells was also suggested to facilitate the growth of the new appendage [114]. Indeed, in the *Xenopus* tail, modulation of environmental ECM changes and apoptosis levels by immune cells allow ROCs to relocalize on the amputation plane to form the specialized wound epidermis [113]. Conversely, mesenchymal cells in the blastema were also shown to express genes associated with the recruitment of immune cells to aid regeneration [115]. Overall, immune cells may influence multiple environmental changes, that are likely to be affecting signalling centres, progenitor/stem cell populations and their interactions to promote regeneration.

Non-immune cells can also influence signalling centres or stem/progenitor cell types to promote regeneration. Secreted factors from nerves can promote proliferation/cell survival required for appendage regeneration in salamanders [116,117], while this phenotype is more developmental stage-specific in the context of *Xenopus* limb regeneration [87,118–120]. Moreover, nerves aid both mouse digit tip and deer antler regeneration, but their elimination does not halt the whole-regeneration programme as seen in salamanders [91,121,122]. By contrast, the abundance of mature chondrogenic and osteogenic cells at the amputation plane is correlated with decreased limb regeneration ability in *Xenopus* [83]. Recently, secreted factors from mature chondrogenic populations, including those that develop into osteoblasts, were found to impair the establishment and maintenance of signalling centre AER cells at the amputation plane, compromising limb regeneration potential [45]. Similarly, when axolotls complete their limb chondrogenesis during their limb development, their limb regeneration fidelity decreases and abnormalities could be seen [95]. This unexpected cell–cell interaction could lead to interesting new hypotheses related to why some animals are regeneration-incompetent. Such as that full limb regeneration ability may be traded with regenerative capacity of the chondrogenic lineage itself or that the pace of chondrogenesis might be the limiting factor determining regenerative success or failure. Furthermore, oxygen levels though the presence/absence of endothelial cells can also impact on the regeneration outcome, as it has been suggested that precocious angiogenesis impairs digit tip regeneration in mice [123]. Overall, cell–cell interactions are likely to be very critical for a regeneration-permissive environment. Such communications can be initiated upon amputation and/or influenced by the homeostatic state of appendages before amputations.

## 5. Conclusion

**Figure 5.**
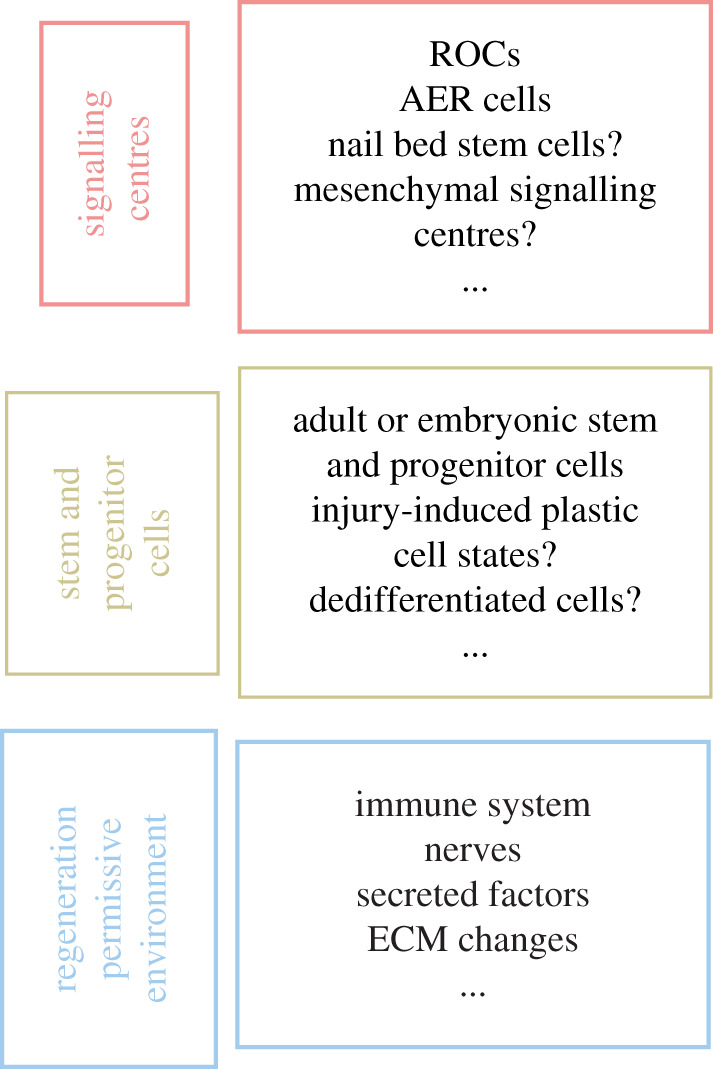
Appendage regeneration paradigms use different cell types and cellular mechanisms; nonetheless, common cellular principles can be found across different appendage regeneration scenarios. Different appendage regeneration paradigms show context dependency at the cellular level. However, in all these paradigms, three common principles could be found: signalling centres, stem and progenitor cell types, a regeneration-permissive environment. Signalling centres orchestrate stem and progenitor cell types to elicit certain functions (e.g. proliferation, migration) in a specific order to regrow the lost appendage. The formation of these cell types as well as their interaction with each other requires a regeneration-permissive environment.

Recent years have provided unprecedented opportunities to decipher the cellular properties of highly heterogeneous tissues by applying cell-centric approaches. Based on these findings, comparisons between paradigms at tissue or gene levels may not be grounded at the cellular level. Nonetheless, appendage regeneration probably involves common features: signalling centres, stem/progenitor cell types and a regeneration-permissive environment (figure 5).

Comparative studies involving the characterization of cell types and cellular mechanisms across species will be required to delineate similarities between regenerative programmes. For this, single-cell-omics approaches will be extremely beneficial in identifying populations as well as the impact of gene activities, including quantitative differences in gene expression levels. Moreover, new insights on cell type diversification and the evolution of regeneration can be obtained through future studies applying these single-cell approaches to different appendage regeneration scenarios in diverse species. For example, cellular maps of regeneration can bring novel perspectives to the big question, if regeneration is an ancestral ability that is lost in certain species or an evolutionary innovation. However, it should be stressed that such powerful single-cell approaches could result in different conclusions depending on the sample size, sequencing coverage and analysis type. Hence, experimental validation and functional analysis of computationally identified potential populations and lineage relations will be required. Not all species-specific phenotypes stem from transcriptional differences. As an example, biochemical properties of proteins have been demonstrated to elicit differences between mammals and guide species-specific developmental programmes [124,125]. From this perspective, cold-blooded aquatic regeneration-competent and warm-blooded terrestrial regeneration-incompetent species would exhibit many differences beyond transcription, such as biochemical, metabolic and biomechanical alterations. Currently, such potential differences between species are heavily underexplored in the context of appendage regeneration. Additionally, it is still quite challenging to test some of the cell-type-specific sub-cellular processes in the context of regeneration (e.g. protein–protein interactions and chromatin modifications). Such sub-cellular studies would delineate how molecular mechanisms aid in regeneration, whether regeneration operates similarly at the molecular level or if the molecular landscape of animals is hardwired differently to respond to amputations.

Another crucial (but very challenging) task will be to decipher the dynamic cellular behaviours and cell–cell interactions necessary for growth and patterning during appendage regeneration. During axolotl digit tip regeneration, the timing of cellular movements was reported to associate with cell-fate decisions, such that early and late migrated fibroblast subpopulations to the blastema were suggested to be more inclined to becoming chondrocytes and fibroblasts, respectively [126]. Such processes are likely to include morphogen gradients and interactions between signalling centres and stem/progenitor cell types. Contemporary advances in explant and stem-cell-based *in vitro* systems allow the study of dynamic properties of cells and cell–cell interactions during development (e.g. somitogenesis [127,128]). Various simplified systems have been incorporated or suggested to study limb or digit tip regeneration: blastema tissue cultures, sliced limb cultures, stem cell-based limb-bud-like progenitor cells and three-dimensional limb-bud-like structures [8,129–136]. Although these methodologies could be helpful to study different aspects of appendage regeneration, dynamic properties and cell–cell interactions may not recapitulate *in vivo* regeneration. Currently, there is no simplified three-dimensional intact limb regeneration system recapitulating *in vivo* early-regeneration stages other than *Xenopus ex vivo* limbs [45,137]. The development of such systems can overcome impractical aspects of *in vivo* research, including high-content live-imaging to tackle dynamics-related questions.

Altogether, advancing to more cell-centric investigations and detailing cellular choreographies for appendage regeneration scenarios in different regeneration-competent species can allow us to screen if similar cell types and mechanisms are also seen in amniotes, and ask what is the cellular recipe for mammalian limb regeneration and where mammals fail at limb regeneration.

## Competing interests

The author declares no competing financial interests.
